# Replacing the Transfusion of 1–2 Units of Blood with Plasma Expanders that Increase Oxygen Delivery Capacity: Evidence from Experimental Studies

**DOI:** 10.3390/jfb5040232

**Published:** 2014-10-27

**Authors:** Amy G. Tsai, Beatriz Y. Salazar Vázquez, Pedro Cabrales, Erik B. Kistler, Daniel M. Tartakovsky, Shankar Subramaniam, Seetharama A. Acharya, Marcos Intaglietta

**Affiliations:** 1 Department of Bioengineering, University of California, San Diego (UCSD), La Jolla, CA 92093, USA; E-Mails: agtsai@ucsd.edu (A.G.T.); pcabrales@ucsd.edu (P.C.); shankar@ucsd.edu (S.S.); mintagli@ucsd.edu (M.I.); 2 Department of Experimental Medicine, School of Medicine, Universidad Nacional Autónoma de México (UNAM), México, D.F. 06726, Mexico; 3 Department of Odontology, Universidad Juárez del Estado de Durango (UJED), Durango, Dgo. 34000, Mexico; 4 Department of Anesthesiology & Critical Care, VA San Diego Healthcare System, La Jolla, CA 92161, USA; E-Mail: ekistler@ucsd.edu; 5 Department of Mechanical and Aerospace Engineering, University of California, San Diego (UCSD), La Jolla, CA 92093, USA; E-Mail: dmt@ucsd.edu; 6 Departments of Cellular and Molecular Medicine and Chemistry and Biochemistry, University of California, San Diego (UCSD), La Jolla, CA 92093, USA; 7 Department of Hematology and Medicine, Albert Einstein College of Medicine (AECOM), Bronx, NY 10461, USA; E-Mail: seetharama.acharya@einstein.yu.edu

**Keywords:** transfusion, oxygen carrying capacity, blood substitutes, oxygen delivery capacity, supra-perfusion, plasma expansion

## Abstract

At least a third of the blood supply in the world is used to transfuse 1–2 units of packed red blood cells for each intervention and most clinical trials of blood substitutes have been carried out at this level of oxygen carrying capacity (OCC) restoration. However, the increase of oxygenation achieved is marginal or none at all for molecular hemoglobin (Hb) products, due to their lingering vasoactivity. This has provided the impetus for the development of “oxygen therapeutics” using Hb-based molecules that have high oxygen affinity and target delivery of oxygen to anoxic areas. However it is still unclear how these oxygen carriers counteract or mitigate the functional effects of anemia due to obstruction, vasoconstriction and under-perfusion. Indeed, they are administered as a low dosage/low volume therapeutic Hb (subsequently further diluted in the circulatory pool) and hence induce extremely small OCC changes. Hyperviscous plasma expanders provide an alternative to oxygen therapeutics by increasing the oxygen delivery capacity (ODC); in anemia they induce supra-perfusion and increase tissue perfusion (flow) by as much as 50%. Polyethylene glycol conjugate albumin (PEG-Alb) accomplishes this by enhancing the shear thinning behavior of diluted blood, which increases microvascular endothelial shear stress, causes vasodilation and lowering peripheral vascular resistance thus facilitating cardiac function. Induction of supra-perfusion takes advantage of the fact that ODC is the product of OCC and blood flow and hence can be maintained by increasing either or both. Animal studies suggest that this approach may save a considerable fraction of the blood supply. It has an additional benefit of enhancing tissue clearance of toxic metabolites.

## 1. Introduction

Blood substitutes research has reached an impasse since the primary motivation for its development no longer has the urgency envisioned in the 80s and 90s when the AIDS epidemic raised concerns about the safety of the blood supply. An additional concern was that trends of the historical use of blood transfusion indicated a continuously increasing demand.

Neither problem has materialized. In terms of transmission of infectious diseases, the blood supply is now as safe, or safer, than it has ever been, with pathogen transmission incidence avoidance at the level of 6 sigma [1]. In terms of intrinsic blood usage, the per capita use in western countries is decreasing as a consequence of technical developments in surgical interventions that minimize blood loss such as robotic surgery [2]. Furthermore, the practice of transfusion is becoming increasingly conservative, *i.e.*, less blood rather than more is transfused.

Conservative transfusion practice is in part due to the increasing awareness that blood transfusion is associated with significant (1 sigma) short and long term (years) morbidity and mortality the latter being directly proportional to the number of units of blood transfused at a given setting [3].

It is notable that to date blood substitutes development has not created a product that in clinical trials has shown an improvement over the use of blood. This outcome suggests that the properties of blood are so far irreproducible and that the effects derived for transfusing blood are not fully understood, *i.e.*, they cannot be identified and implemented by alternative approaches available today.

Blood transfusion focuses on increasing blood’s oxygen carrying capacity (OCC), which is assumed to be a marker that insures that organs and tissues receive an adequate oxygen supply. This is an elusive goal to achieve, by transfusing blood or blood substitutes, because the effects from transfusion are only partially related to the increase in intrinsic OCC; the desirable functional effect is the restoration of circulatory oxygen delivery capacity (ODC), which is the product of blood flow (perfusion) and OCC.

It should be apparent that products based on molecular solutions of chemically modified hemoglobin (Hb) have an inherent handicap, since there does not appear to be a practical way to eliminate scavenging of nitric oxide (NO) which is the cause of vasoconstriction and reduction in blood flow. This effect counteracts the significant lowering of blood viscosity and peripheral vascular resistance due to anemia, which in itself also decreases NO bioavailability owing to the associated decrease in circulatory shears stress which lowers the production of NO by mechanotransduction, thus limiting the bioavailability of this vasodilator.

These considerations lead to questioning the critical need for blood substitutes, beyond the obvious need for a suitable surrogate when blood is unavailable. Additionally, it is instructive to relate hypothetical or factual needs with what can be realistically accomplished with current state of the art understanding of the functional effects of anemia, and how these can be addressed by proposed blood surrogates. This will form the theme for next sections.

## 2. The Need for a Blood Substitute

Blood transfusion is generally considered to be a safe and effective procedure, a concept that is now increasingly challenged. It is lifesaving in massive blood losses, and it improves the quality of life, ameliorating symptoms of malaise, in individuals with chronic anemia [4]. However, statistics show that it causes side effects in 10% of transfusions, and serious side effects in 1/5000 transfusions, primarily due to transfusion-related acute lung injury (TRALI). This is reported to presently cause mortality in 10–20 individuals in the US per year, a number that may be underreported [5]. It is doubtful that a drug with this safety profile would be approved for human use today, when societal safety expectation is in the range of 6 sigma. Shander *et al.* summarized in a comprehensive review the relative dangers of anemia and its treatment by blood transfusion showing the difficult choices that must be made when the acute restoration of oxygenation is necessary [6].

These statistics pale when considering the long term (years post transfusion) effect of transfusing a unit of blood. Many retrospective studies show that transfusing one unit of blood after a successful surgical intervention correlates with a 6% increase in mortality 10 years later. Furthermore, this effect appears to be directly proportional to the number of units transfused, up to about 3–4 units, and is therefore associated with a mortality increase to up to 20%–30% at 10 years post-transfusion [7,8].

Blood transfusion is usually called for when Hb falls below circa 7 g/dL in patients, with higher Hb thresholds with advancing age. As a consequence about 1/3 of the world blood supply is transfused under these criteria, although the physiological effects of the intervention are not well understood, and the rationale for this intervention appears to reside in the anecdotal evidence that patients “feel good”. The 1/3 number is probably an underestimate since data from hospitals reporting specific information show higher proportions [9]. As an example the state of Western Australia, known for its meticulous management and motoring of the blood supply, reports the use of 14.4% and 43.7% of the total blood supply in 1 and 2 units transfusions [10]. A study of blood use in 18 hospitals in Austria showed respectively the use of 13% and 56% of the total blood supply [11].

In practice, two units of blood are often transfused when one unit of blood is considered sufficient. However, transfusion of a unit of blood does not treat anemia, considering the proposition, “the patient whose requirements are met with one RBC unit is no more in need of a transfusion than the donor who gives 500 mL of blood”. [12].

Transfusion of limited units of blood is prescribed by a combination of factors where the most significant one is the blood Hb content, which defines the so called transfusion trigger. A 50% reduction in blood Hb is a nominal value indicating the need for transfusion, which varies according to the age of patient and his/her clinical condition. Hospital guidelines, clinical experience, and more recently the awareness of the need to minimize allogeneic blood exposure are additional factors that contribute to setting the transfusion trigger. Single unit blood transfusion is increasingly recommended as studies show that the clinical outcome is not different from two unit transfusions [13]. Independent of this consideration, the literature shows that 1–2 unit transfusions are common and associated with worse outcomes after cardiac operations. In the absence of a mechanistic justification this level of transfusion appears to be discretionary, hence avoidable, especially in view of the many studies that document its association with worsening outcomes [14].

Whether the transfusion of 1–2 units of blood increases tissue oxygenation is not clear cut and there is evidence that in some cases it may cause a decrease [15], an effect that could be related to blood storage effects, the increase in blood viscosity and inflammatory and immunological reactions.

These considerations suggest that there is a real, substantial and imminent need for the development of a blood substitute that specifically targets substituting the transfusion of 1–2 units of blood under the blood transfusion paradigm where the increase in intrinsic blood OCC does not appear to be the critical issue. Furthermore, for patients deemed to need an improved oxygen delivery, the objective of transfusing 1–2 units of blood could in principle be obtained with procedures that increase ODC, *i.e.*, perfusion rather than OCC.

## 3. Blood Substitutes Targeting 1–2 Blood Transfusion

Present trends in blood substitutes development appear to have shifted emphasis from attempting to develop products for treating massive blood loss to fluid formulations that target specific anoxic regions, simultaneously “treating” the potential dysfunctions due to the localized hypoxia [16]. This approach, in principle requiring comparatively small volume infusions, is based on the realization that perfusion with a Hb solution chemically modified to induce a very high oxygen affinity, *i.e.*, a left shifted oxygen dissociation curve, would unload oxygen only in areas with very low tissue oxygen. Polyethylene glycol (PEG) conjugated Hb, labeled MP4, a product whose commercialization was attempted by Sangart Inc. (San Diego, CA, USA) had this characteristic [17].

The possibility of adding compounds that could in principle limit the toxicity due to tissue hypoxia, such as the generation of reactive oxygen species, promoted the formulation of “oxygen therapeutics”. These materials in combination with the concept of targeted oxygen delivery serve to treat the effects of tissue re-oxygenation (reperfusion injury).

The group of Simoni *et al.* developed an Hb-based oxygen carrier that included ATP-adenosine and glutathione (GSH) cross-linked to Hb (ATP-ADO-GSH-Hb). The product is commercialized under the name of HemoTech, by HemoBioTech Inc. (Dallas, TX, USA). This material provides oxygen delivery while controlling vasoconstriction, oxidative stress, and inflammation due to the presence of Hb in solution in blood. Remarkably, it stimulates erythropoiesis [18]. This material also shields heme from reactive oxygen species and introduces an electronegative charge onto the surface of Hb, reducing renal excretion, extravasation, and phagocytosis. However, the specific effect on blood OCC and ODC does not appear to be reported for either experimental or clinical studies, and most of its activity is reported in term of biochemical and cellular effects, rather than functional effects.

SynZyme Technologies LLC (Irvine, CA, USA) developed the multifunctional product polynitroxylated pegylated Hb (PNPH) that transports oxygen, is an antioxidant and provides for plasma expansion properties due to pegylation [19]. It combines Hb and Tempol (4-Hydroxy-2,2,6,6-tetramethyl-piperidine-*N*-oxyl), an agent that reduces blood pressure in hypertensive rodent models and mitigates endothelial dysfunction. Tempol is a free radical scavenger which mitigates the effects of excessive superoxide production and NO scavenging arising from the presence of acellular Hb in the circulation.

Sangart Inc. (San Diego, CA, USA) introduced CO-MP4 comprised of the Hb-based oxygen therapeutic agent MP4 bound to carbon monoxide (CO), as a means to deliver CO, and reported that delivered CO from the circulation reduced ischemia/reperfusion injury in rats, providing the first evidence that MP4 is a CO therapeutic agent [20]. However, the rationale for using a Hb based carrier such as PEG-Hb, that scavenges NO, to deliver CO is not clear since administering CO dissolved in saline has the same effect and does not appear to have been considered [21].

The question whether these materials would be physiologically relevant and cost effective in substituting transfusion of 1–2 units of blood should be posed since there is scant physiological or clinical evidence supporting this contention. An important question is the concentration they can achieve in blood. Experimental studies show that when an area of tissue is anoxic, arteriolar as well as capillary blood is under-oxygenated, and furthermore capillaries in general do not supply much oxygen to the tissue [22]. Normalizing the oxygen supply to arterioles and the capillaries in ischemic tissue (in a 50% anemic individual, *i.e.*, 7 g Hb/dL) requires increasing OCC of blood by about 3 g Hb/dL or more depending on the level of ischemia of the target organ, which implies introducing about 150 g of Hb into the circulation when using molecular Hb-based blood substitutes, a level that in most cases leads to several toxicities [23,24].

In summary, the present re-direction of blood substitutes development into “therapeutic” agents appears to be saddled by the recurrent theme that the principal objective is the restoration of OCC, whose fulfillment requires using Hb, a material of high intrinsic toxicity once outside of red blood cells (RBCs). Thus, a substantial component of the therapeutic effect of these compounds may be directed at treating or mitigating their inherent toxicity due to Hb [25]. Furthermore, it is notable that none of these compounds has been tested so far in studies of microvascular function, a standing FDA recommendation.

## 4. Treating Anemia by Increasing OCC with Limited RBC Unit Transfusion

The practice of transfusion rests on the two fundamental principles that cardiovascular function requires the restoration of OCC, and that low blood viscosity is always to be preferred to high blood viscosity. Engineering studies of the circulation at UCSD during the past 3 decades show that while these principles are true, their application presents such a wide latitude that, the exceptions may account for the wrong approach and negative outcomes in as many as half of all transfusion interventions.

The cardiovascular system is designed to supply the materials necessary to sustain tissue metabolic rate. This is a rate, and therefore for all components of this process it implies the product of the concentration of the material in the supply stream times the flow velocity of the stream, the relative proportions of these factors not being relevant.

Blood flow rate is determined by the pressure imparted by the heart and the hydraulic resistance of the circulation. The hydraulic resistance of the circulation, in the absence of vasoactive affects, depends on the viscosity of blood, which is a strong nonlinear function of RBC concentration (hematocrit, Hct). In principle blood loss decreases Hct and therefore blood viscosity, and blood flow compensates the decrease of OCC by increasing blood flow velocity thus maintaining ODC, a zero sum game. However the non-linearity of the blood viscosity-Hct relationship breaks down in the microcirculation, where Hct is about half of the systemic and diluting Hct and has little effect on local blood viscosity. This is a well-known effect due to isovolemic hemodilution, investigated and documented since the 1970s by the studies of Mesmer *et al.* [26,27].

A critical factor (known since the 1990s) is that the microcirculatory lack of response to the effects of hemodilution is overcome by increasing plasma viscosity as shown by experimental studies of Tsai *et al.* [28,29,30,31]. Their results, supported by sporadic previous studies from other laboratories [32,33], show that the missing element in hemodilution is the maintenance or increase of capillary and microvascular shear stress that according to the tenets of mechanotransduction leads to increased NO production since endothelial shear stress is directly proportional to local blood viscosity. This effect induces vasodilatation and supra-perfusion, a fundamentally new parameter in resuscitation.

During hemodilution the increased flow in the circulation in response to decreased blood viscosity appears to be insufficient to maintain endothelial shear stress and the production of vasodilators. According to Kameneva *et al.* [34], the dependence of blood viscosity on Hct is nonlinear and blood viscosity and therefore vascular resistance falls about twice as fast as Hct in an individual with normal Hct. This should correspond to a proportionate, compensatory increase in blood flow, an effect that occurs only over a small range of Hct decreases, not evident in 50% anemia.

Changing Hb concentration (*i.e.*, Hct) in an anemic person causes changes in systemic blood viscosity directly proportional to changes in Hct. Furthermore endothelial shear stress is directly proportional to the local blood viscosity, being maximal in arterioles and capillaries (order of magnitude greater than that of all other blood vessels [35]). However Hct in arterioles is already significantly decreased from systemic values in normal conditions, having an intermediate value between capillaries and central blood vessels. As a consequence, changes in Hct in an anemic patient, induced by 1–2 units blood transfusion do not appear to translate into the significant restoration of arteriolar blood viscosity and shear stress.

In summary, the distribution of Hct in the circulation, the nonlinear relation between blood viscosity and Hct, and the non-uniform distribution of endothelial shear stress and its role in regulating vasoactivity mandate that lowering blood viscosity by decreasing Hct results in decreased blood flow and perfusion. This appears to be due to a shear stress stimulus deficit that prevents vasodilatation, which explains why increasing microvascular shear stress in hemodilution induces supra-perfusion, re-establishing the ODC that should be inherent to hemodilution.

In terms of blood substitutes-based molecular Hb solutions, it should be apparent that their lack of viscogeneicity and their lingering vasoconstrictive properties inherent to Hb in solution in blood promote conditions that fundamentally hinder the goal of supplying oxygen to the tissue, particularly if used under the umbrella of “oxygen therapeutics”.

## 5. Treating Anemia by Increasing ODC

In terms of mechanics it should be possible to “trick” the circulation into providing the necessary oxygen to the tissue by trading increased flow velocity with lowered OCC or Hct since this lowers blood viscosity. The experimental data suggests that this is possible by manipulating the viscosity of plasma, which unlike the viscosity of blood is uniform throughout the circulation.

This is shown by the experimental results of Tsai *et al.* and Cabrales *et al.* who reversed the shear stress deficit in extreme hemodilution by using hyperviscous plasma expanders, namely colloidal solutions dextran of 500 kDa [36] and alginates [37]. These findings demonstrate that hyperviscous plasma expansion resuscitation in experimental models is effective in treating extreme anemia [37,38] and hemorrhagic shock [29,38,39].

Strategies and materials for increasing plasma viscosity are not entirely risk-free since large molecular species in plasma have a tendency to cause RBC aggregation, which is promoted by most plasma hyperviscosity-inducing compounds based on high molecular weight colloids, such as high molecular weight Dextran, hydroxyethyl starch (MW > 500 kDa), and alginates. However this effect is also a function of Hct, and therefore it is minimized in extreme hemodilution.

PEG-Alb was developed at Enzon Inc. (Piscataway, NJ, USA) [40], and was re-developed by Sangart Inc., San Diego, to serve as a non-oxygen carrying control for PEG-Hb that could be used to compare the physiological behavior due to oxygen carrying *vs.* non-oxygen carrying [41] resuscitation fluids based on otherwise identical solution properties (viscosity, COP and nearly comparable hydrodynamic volume of PEGylated protein) when used in small dosages.

PEG-Alb is a compound of moderate bulk viscosity that is a strong inductor of the supra-perperfusion effect. Its effectiveness is due to inducing non-Newtonian blood rheological behavior causing anemic blood to increases its shear thinning properties, leading to increased production of vasodilators by mechanotransduction as shown by direct measurements of NO in the microvascular wall [42]. The viscosity of 4% PEG-Alb is 2.4 cP (37°) normal human plasma is ~1.2 cP [43] and 5% human serum albumin is 0.9 cP, while normal blood viscosity is in the range of 4.5 to 6.0 cP depending on Hct.

It should be noted that increased perfusion in general benefits improving tissue washout, being one of the treatments of endotoxemia and sepsis. In this context we have shown experimentally that supra-perfusion induced by PEG-Alb based fluid treatment of LPS-induced endotoxemia was superior to other forms of fluid treatment in aiding recovery of microvascular function [44].

It should be noted that we do not refer to tissue oxygen distribution as a parameter for evaluating survival. This is not a determinant factor in the limiting conditions, where most of the supplied oxygen is used by the tissue, which leads to essentially a zero oxygen partial pressure in the tissue. Many studies from our laboratory have shown that the most critical functional marker of tissue and whole organism survival in extreme anemia and hemorrhage is functional capillary density (FCD) [45].

## 6. Maintenance of the Oxygen Metabolic Requirements

Among the many ways to insure adequate ODC, an option is to reduce oxygen demand, which increases oxygen availability. Such a phenomenon should be intrinsic to our proposed form of plasma expansion which we find to be based on the increase of NO bioavailability, *i.e.*, a gas that controls tissue oxygen demand. This is shown by the relation between NO levels and oxygen demand. An example of this phenomenon is that NO formation inhibition increases resting muscle oxygen uptake by 20% in studies carried out in conscious dogs at rest and during exercise. These studies show that inhibition of NO synthesis increases O_2_ consumption independently of changes of skeletal muscle blood flow [46], while cell respiration is inhibited when inducible NO synthase increases NO generation [47].

The biochemical evidence that increased NO bioavailability leads to decreased oxygen consumption and therefore decreases the need to maintain increased oxygen supply is superseded by simple biomechanical effects due to the structure of oxygen transport in the circulation. In essence, the heart imparts pressure to blood which is propelled through the circulation, devoid of any barrier to prevent the exit of oxygen prior to its arrival to where it is needed.

The lack of a specific barrier to the diffusion of oxygen out of the blood vessels, and the high oxygen concentration (*i.e.*, oxygen partial pressure or pO_2_) of arterial oxygen relative to tissue pO_2_ of the surrounding tissue causes a significant oxygen flux out of the arterial circulation (this being is in part the reason why hyperoxic oxygenation is not as effective as expected). This oxygen loss is mediated by the period that blood is in transit between lungs and tissue microcirculation and therefore blood flow velocity [48]. This is a mathematically complex problem; however simplified solutions [49] show that changes in the rate of oxygen delivery are approximately similar to changes in blood flow velocity. Consequently, significant changes in tissue oxygenation can be obtained by manipulating hemodynamics rather than OCC.

## 7. Experimental Basis

The experimental evidence leading to our tenet that OCC can be replaced with the increase of ODC originates in the experimental work of Tsai *et al.* [30] who investigated the microvascular responses in extreme hemodilution in the hamster window chamber model that allows the study of microcirculation in an intact tissue, isolated from the environment and without anesthesia [50,51]. The significance of this approach is that the biological and transport properties of blood interact with the tissue in the microcirculation, a process that ultimately manifests itself at the systemic level. However, without microvascular information, it is practically impossible to interpret the systemic effect due to anemia, blood transfusion and the introduction of fluids that change blood composition. Notably, to date, there is no attempt to investigate “oxygen therapeutics” from the perspective of their effects in the microcirculation.

In particular, the microcirculatory experimental approach allows evaluation of the tissue responses to the local level of perfusion, microvascular wall NO concentration and local pO_2_, and the critical parameter functional capillary density (FCD) that is not accessible to measurement in clinical and most experimental physiology approaches [52]. The importance of this parameter was demonstrated in studies of hemorrhagic shock, which showed that survival is primarily determined by the maintenance of FCD, and not tissue oxygenation [45,52].

Microvascular experimental studies showed that there is a special type of plasma expander that by increasing plasma viscosity induces greater shear stress on the endothelium, an effect that leads to the liberation of vasoactive substances, such as NO, prostaglandins, *etc.*, that cause the supra-perfusion effect due to vasodilatation [37]. In the absence of vasodilatation blood transfusion in most cases is a zero sum game in term of increasing ODC since the increase in blood viscosity, which occurs prevalently in the systemic circulation, lowers flow and eliminates the effect of increased OCC [53]. The determining role of blood viscosity in tissue survival was demonstrated in studies of hemorrhagic shock that showed that the same resuscitation effect was obtained with blood transfusions where the added OCC was eliminated by saturating the transfused blood with carbon monoxide [54] or using viscogenic plasma expanders instead of blood [55].

The development of PEG-Alb provides additional support to the concept that viscosity and its ability to induce supra-perfusion yields the same benefit as the increase in OCC. Studies comparing PEG-Alb and PEG-Hb in extreme hemodilution [41] and hemorrhagic shock [56] showed there were no critical differences in outcome associated with the difference in OCC between solutions. However, in terms of viscogeneicity, PEG-Alb was superior to other viscogenic plasma expanders because the induced changes in blood viscosity due PEG-Alb did not adversely influence heart function [57,58,59]. This effect was subsequently explained by the studies of Sriram *et al.* [42] who showed that it is due to the shear thinning properties that PEG-Alb induces in blood.

## 8. Etiology of Anemia: Is There a Uniform, Universal Palliative Treatment?

A critical problem in devising an approach to the treatment of anemic conditions are the physiological differences between an organism where anemia is an acute problem due to hemodilution or hemorrhage, and the setting of chronic anemia induced by disease processes. These conditions evoke different forms of adaptation and protective responses that logistically should benefit from targeted therapeutic approaches.

The experimental data from most studies addressing the problem of restoring ODC have exclusively dealt with restoring OCC to treat the acute problem, and to our knowledge there are no experimental studies on the effects of transfusing blood, or blood substitutes in animal models (including our studies) with long term adaptation to anemia. However, to date, when the immediate clinical need arises to treat acute or long term anemia the universal approach is increasing OCC by blood transfusion, a procedure that solves the acute problem, but has statistically predictable, not preventable incidence of high mortality.

The approach suggested by experimental results, which works in different forms of acute anemia, is based on fundamental physical transport considerations and basic physiological mechanisms. Metabolism consumes oxygen, which is a rate of oxygen utilization. This can only be maintained by a matching rate of oxygen supply, which comprises carrier capacity and speed of delivery. Clearly augmenting delivery rate sustains the oxygen supply, which in experimental studies is effective in acute situations. Therefore, a question to explore is whether this approach can be used in organisms adapted to anemia.

## 9. Conclusions

Neither blood substitutes nor plasma expansion development have produced major results in the clinic in the past decade. Furthermore these fields have remained compartmentalized, although it should be apparent that an oxygen carrying blood substitute should also have “good” plasma expansion properties, inducing circulatory conditions that aid blood’s oxygen delivery and equally importantly the maintenance of vessel wall shear stress.

Evolution of blood substitutes development into the formulation of “oxygen therapeutics” is a potential new outcome, however, at present it would seem that their primary effect is that of overcoming their auto-generated toxicity, since they are based on molecular Hb-based compounds in solution.

We propose that a missed opportunity that should be actively explored is the development of products devoid of auto-toxicity, useful in restoring blood volume and increase ODC in situations not associated with massive blood losses, when the transfusion of 1–2 units of blood is deemed necessary. This approach could have a significant therapeutic effect, also aided by the inherent increase of tissue washout of potentially toxic metabolites and CO_2_.

Our hypothesis and results suggest that supra-perfusion resuscitation fluids could be used in the acute treatment of patients with acute anemia secondary to hemorrhage or critical anemia due to other pathologies, and possibly in the acute treatment of patients with hemoglobinopathies and associated anemias (*i.e.*, sickle-cell, *etc.*), bringing oxygen to hypoxic regions in conjunction with increased perfusion. Furthermore, introduction of this approach could significantly improve the quality of the blood supply, since the decrease of a potentially significant fraction of blood transfusions would allow shorter storage times, a factor in blood transfusion effects on short and long term morbidity.
